# Association between TikTok Addiction and Anxiety, depression, and Instagram Addiction among Tunisian University Students

**DOI:** 10.1192/j.eurpsy.2026.11313

**Published:** 2026-07-31

**Authors:** S. Ben Fredj, R. Ghammam, A. Skhiri, N. Zammit, O. Ben Saad, A. Chouchen, I. Harrabi

**Affiliations:** 1 Department of Preventive Medicine and Epidemiology, “LR19SP03”, University Hospital Farhat Hached; 2 Faculty of Medicine of Sousse, University of Sousse; 3 Department of Occupational Medicine, “LR19SP03”, University Hospital Farhat Hached, Sousse, Tunisia

## Abstract

**Introduction:**

The rapid global proliferation of social media, including the TikTok platform, has fundamentally altered communication and information consumption patterns, particularly among young adults. While offering benefits like connectivity and learning, this digital engagement has also raised significant public health concerns regarding its addictive potential. TikTok addiction is increasingly associated with adverse psychological outcomes, including anxiety, depression, and addiction to other Problematic Social media Use.

**Objectives:**

To determine the association between TikTok addiction and mental health disorders such as anxiety, depression, Instagram addiction, and Facebook addiction, among Tunisian university students

**Methods:**

A cross-sectional study was conducted among university students from October to November 2023. For data collection, we used an online structured questionnaire gathering sociodemographic characteristics, mental health disorders, Tikok, and other social media addiction. We used a modified Bergen scale from Facebook to assess TikTok and Instagram addiction. Problematic Facebook use was assessed using the Arabic version of the Bergen Scale The Hospital Anxiety and Depression Scale (HADS), validated in Arabic, was used.

**Results:**

In total, 326 students answered the questionnaire. The majority (71.5%) were female, and the average age was 23.06±3.03. In terms of educational paths, 57.1% of participants were undergraduate (1^st^ academic level), 57.1% were graduate (2nd academic level), and 19% were postgraduate. The prevalence of TikTok addiction increased with mental health disorders. A statistically significant relationship was observed between TikTok addiction and anxiety (16.3% vs 11.1% vs 3.8% in participants who had severe symptoms, moderate symptoms, and no symptoms of anxiety, respectively; p=0.006). Interestingly, the mean scores for Instagram addiction were found to be highest among university students addicted to TikTok compared to those with problematic use of TikTok.

**Conclusions:**

Although TikTok use seems to be an inherent characteristic of modern life, regardless of geographic location, culture, socio-economic level, or education, there is a risk of behavioral addiction linked to its abuse, which could harm the mental health of users. Conversely, it remains unclear whether anxiety and depression elevate an individual’s risk of developing TikTok addiction or the TikTok addiction increases the likelihood of these disorders. Thus, warranting comprehensive longitudinal studies that examine the bidirectional relationships between these mental health variables over time.

**Disclosure of Interest:**

None Declared

